# Alternative approaches for studying humanitarian interventions: propensity score methods to evaluate reintegration packages impact on depression, PTSD, and function impairment among child soldiers in Nepal

**DOI:** 10.1017/gmh.2015.13

**Published:** 2015-08-12

**Authors:** B. A. Kohrt, M. Burkey, E. A. Stuart, S. Koirala

**Affiliations:** 1 Transcultural Psychosocial Organization (TPO) Nepal, Kathmandu, Nepal; 2 Duke Global Health Institute and Department of Psychiatry and Behavioral Sciences, Duke University, Durham, NC, USA; 3 Division of Child and Adolescent Psychiatry, Johns Hopkins School of Medicine, Baltimore, MD, USA; 4 Departments of Mental Health, Biostatistics and Health Policy & Management, Johns Hopkins Bloomberg School of Public Health, Baltimore, MD, USA

**Keywords:** Children and adolescents, intervention, post-traumatic stress disorder (PTSD), statistical methods, war

## Abstract

**Background.:**

Ethical, logistical, and funding approaches preclude conducting randomized control trials (RCTs) in some humanitarian crises. A lack of RCTs and other intervention research has contributed to a limited evidence-base for mental health and psychosocial support (MHPS) programs after disasters, war, and disease outbreaks. Propensity score methods (PSMs) are an alternative analysis technique with potential application for evaluating MHPS programs in humanitarian emergencies.

**Methods.:**

PSMs were used to evaluate impacts of education reintegration packages (ERPs) and other (vocational or economic) reintegration packages (ORPs) *v.* no reintegration programs on mental health of child soldiers. Propensity scores were used to determine weighting of child soldiers in each of the three treatment arms. Multiple linear regression was used to estimate adjusted changes in symptom score severity on culturally validated measures of depression, post-traumatic stress disorder (PTSD), and functional impairment from baseline to 1-year follow-up.

**Results.:**

Among 258 Nepali child soldiers participating in reintegration programs, 54.7% completed ERP and 22.9% completed ORP. There was a non-significant reduction in depression by 0.59 (95% CI −1.97 to 0.70) for ERP and by 0.60 (95% CI −2.16 to 0.96) for ORP compared with no treatment. There were non-significant increases in PTSD (1.15, 95% CI −1.55 to 3.86) and functional impairment (0.91, 95% CI −0.31 to 2.14) associated with ERP and similar findings for ORP (PTSD: 0.66, 95% CI −2.24 to 3.57; functional impairment (1.05, 95% CI −0.71 to 2.80).

**Conclusion.:**

In a humanitarian crisis in which a non-randomized intervention assignment protocol was employed, the statistical technique of PSMs addressed differences in covariate distribution between child soldiers who received different integration packages. Our analysis did not demonstrate significant changes in psychosocial outcomes for ERPs and ORPs. We suggest the use of PSMs in evaluating non-randomized interventions in humanitarian crises when non-randomized conditions are not utilized.

## Background

A challenge in global mental health research is identifying research methods and statistical techniques to evaluate interventions in settings that may not be amenable to randomized control trials (RCTs). For example, RCTs may be difficult to implement in humanitarian crises, such as environmental disasters, political violence, and infectious disease outbreaks. Only a few RCTs have been conducted in humanitarian crises, and they have tended to focus on adults or have examined interventions that are not widely used in humanitarian crises (Tol *et al.* 2011); moreover, Tol and colleagues observed that the most frequently used interventions in humanitarian crises are unfortunately the least studied approaches. Ethical issues related to appropriateness of waitlist conditions and lack of acceptable treatment-as-usual alternatives may preclude conducting an RCT (Allden *et al.* 2009). Logistical issues related to blinding, randomization, and intervention contagion are also challenges for RCTs in humanitarian crises. In addition, there are limited funding mechanisms that can rapidly make research grants available to study mental health and psychosocial in these settings.

These factors have contributed to a lack of intervention research in humanitarian crises, leaving the field with a limited evidence-base for best practices to promote mental health and psychosocial well-being (Tol *et al.* 2011). Because humanitarian crises disproportionately affect low- and middle-income countries (LMICs) (Tol *et al.* 2011) and children in LMICs bear the greatest burden of crises such as armed conflict (United Nations, 2014), addressing this gap between research and practice is an important area for global mental health.

### Propensity score methods (PSMs) to evaluate humanitarian interventions

In context where it is not ethical or feasible to implement RCTs, one alternative is to use statistical methods to evaluate the impact of intervention components in contexts where a population is given non-randomized access to services. For example, play-interventions, safe spaces, and other activities are commonly provided to children in humanitarian psychosocial responses. In most contexts, these services will not be used by all children and families or at least not used to the same degree. While statistical approaches can be used to compare outcomes between children who did and did not use these services, there are likely multiple confounds that influence which children and families use these services; e.g. either more distressed or less distress children may be using the services thus precluding general comparison of mental health after participating in such an intervention.

PSMs are a set of statistical techniques that can be employed in such contexts because they can account for observed potential confounds between children who are low and high service users. PSMs provide an estimate of treatment effects in non-randomized intervention trials by identifying a control group that is similar to the intervention group with respect to the observed covariates (Rosenbaum & Rubin, 1985). The technique yields treatment and control groups that have similar distributions of observed covariates, thereby approximating one of the fundamental benefits of RCTs in an observational study (Stuart, 2010).

PSM approaches proceed using a two-step analytic approach: first, data are preprocessed (without reference to outcome data) in order to match or weight participants on a combination of observed covariates, with the aim of reducing the association between the treatment assignment and confounding variables. The method assumes that this preprocessing minimizes differences between treatment and control groups on factors that would be expected to affect outcomes (i.e. confounders). Second, after the propensity score model is finalized, matches and/or weighting are incorporated into outcome models as appropriate.

In addition to the philosophical appeal of separating decisions about controlling for confounding from outcome model development, PSMs have several advantages over standard regression techniques (Stuart, 2010): first, PSMs identify and help to account for areas in which covariate distributions do not overlap sufficiently. Insufficient overlap often leads to extrapolation and poor performance of standard regression techniques. Second, PSMs include more straightforward diagnostic approaches to check model assumptions. Third, PSMs can be used in combination with and improve the performance of other regression models. Finally, standard regression relies heavily on correct specification of the underlying model (of confounding), whereas PSMs are more flexible in the typical case in which the exact relationships between confounders and treatment assignments are unknown.

Therefore, PSMs have promise as a tool to improve evaluation for humanitarian programs. There is a growing precedent for the use of PSMs to evaluate ‘natural experiments’, e.g. the effects of remittances on disaster preparedness (Mohapatra *et al.* 2012), and domestic and international policies, e.g. the effects of foreign aid on civil conflict intensity (Strandow, 2014). PSMs have been used to evaluate planned interventions in humanitarian crises, such as food supplementation for children in Niger (Isanaka *et al.* 2010). In relation to demobilization and reintegration humanitarian programs for former combatants, studies in Burundi and Sierra Leone have employed PSMs to evaluate economic impacts (Humphreys & Weinstein, 2007; Gilligan *et al.* 2013). PSMs have also been used to assess political, economic, and psychological consequences of child soldiering in Uganda (Blattman & Annan, 2010). In this paper, we apply PSMs to evaluate the impact of reintegration packages on child soldier mental health and psychosocial well-being in Nepal.

### Gaps in evaluation of child soldier reintegration research

Child soldiers, also known as children associated with armed forces and armed groups, are defined as any person below 18 years of age who is or who has been recruited or used by an armed force or armed group in any capacity, including but not limited to fighters, cooks, porters, messengers, spies, or for sexual purposes. It does not only refer to a child who is taking or has taken a direct part in hostilities (UNICEF, 2007, p. 7).

Child soldiers commonly have adverse psychological and psychosocial outcomes following involvement in armed conflicts (Betancourt *et al.* 2013). In studies using comparison groups, the burden of mental health and psychosocial problems is greater among child soldiers than among civilian children experiencing the conflict (Okello *et al.* 2007; Kohrt *et al.* 2008; Blattman & Annan, 2010; Betancourt *et al.* 2011). In addition to greater exposure to traumatic wartime events, child soldiers often experience rejection and discrimination from families, teachers, peers, and other community members (Betancourt *et al.* 2013).

The standard practice for international non-governmental organizations (NGOs) and other institutions involved in humanitarian assistance for child soldiers is to conduct disarmament, demobilization, and reintegration (DDR) activities (Williamson, 2006; Humphreys & Weinstein, 2007; Annan *et al.* 2009). There are many schools of thought about the best approaches to improve psychosocial well-being and mental health among former child soldiers in DDR programs. For example, some practitioners and advocates have argued against mental health terminology and psychotherapeutic interventions because of concerns that they may be stigmatizing, unhelpful, and potentially harmful in non-Western cultural settings (Bracken *et al.* 1996; Bracken & Petty, 1998). Others have argued for the need to assure availability of psychiatric and psychological services because these have a strong evidence-base (in high resource settings) for reducing distress (Dyregrov *et al.* 2002). The majority of the field, however, calls for a combination of services ranging from assuring basic economic and livelihood needs to community-based psychosocial care to specialized mental health services (Betancourt & Khan, 2008*a*; Betancourt & Williams, 2008*b*), as exemplified by the Inter-Agency Standing Committee (IASC) Intervention pyramid for mental health and psychosocial support (MHPS) in emergencies (IASC, 2007, p. 12).

Unfortunately, there is a dearth of evidence on the mental health impact of these different types of services in humanitarian settings. Although there have been randomized trials of specific interventions for child soldiers (Ertl *et al.* 2011) and other children affected by war (Jordans *et al.* 2009), the most commonly used general psychosocial services and reintegration support models have received little evaluation (Tol *et al.* 2011). The theory of change for education, economic, and social programs is that returning to normal activities will allow children to undergo natural processes of recovery. Moreover, education, economic, and social programs are believed to improve mental health by increasing likelihood of access to basic needs and other resources required for survival and well-being.

However, research on educational, economic, and social reintegration programs during DDR is lacking to determine if and how they impact mental health. From the studies that have been conducted, the benefits of some DDR programming is variable. For example, in Burundi, a retrospective tracer approach was used to compare child soldiers receiving socioeconomic reintegration support with civilian children, which revealed no differences in the groups approximately 3 years after the reintegration support (Jordans *et al.* 2012). In contrast, a prospective study in Nepal among child soldiers (referred to as verified minors) failed to identify a contribution of reintegration packages to changes in mental health, with the exception that vocational reintegration packages predicted *poorer* mental health outcomes in the domain of post-traumatic stress disorder (PTSD) symptoms (Adhikari *et al.* 2014). However, these studies are limited by potential confounds in the design and analysis.

PSMs can address some of these limitations. Therefore, the goal of this study was to address the gap in research literature regarding benefits of reintegration packages on mental health. We used PSMs to study the impact of reintegration packages *v.* no treatment on psychosocial and mental health outcomes of child soldiers in Nepal.

## Methods

### Setting: child soldiers in Nepal

Nepal is a landlocked country north of India and south of the Tibetan autonomous region of China, with a population of approximately 28 million. Nepal is a low development country with a rank of 157 out of 186 countries, a life expectancy of 69.1 years, a mean of 3.2 years of education, and a gross national income per capita of US$1,137 (UNDP, 2013). Nepal's population comprises more than 60 ethnic and caste groups (Whelpton, 2005). The society traditionally has been categorized according to a Hindu caste system with *Bahun* (priest caste) and *Chhetri* (warrior/royalty caste) at the top of the hierarchy. *Dalit/Nepali* (untouchables) are the lowest strata of castes. Other ethnic groups (referred to as *Janajati*), which include other Hindu groups, Buddhists, Muslims, and Kirantis, have different positions in the social hierarchy based on their degree of Hindu purity. The caste stratification influences society heavily with *Bahun* and *Chhetri* dominating institutions of government, education, and business (Kohrt, 2009). *Dalit/Nepali* castes bear the greatest burden of common mental disorders in adult community populations (Kohrt *et al.* 2009).

In 1996, the Communist Party of Nepal [Maoists, CPN (M)] presented demands to the government of Nepal to address economic and social injustices, abolish the monarchy, end caste- and gender-based discrimination, and establish a constituent assembly. When the government refused to address these demands, the CPN (M) began an agrarian revolution. Government security forces and Maoists killed over 17,000 people during the People's War, which lasted 11 years; with the majority of deaths at the hands of the Royal Nepal Army and the government's police force (INSEC, 2008). The war ended in November of 2006, when the CPN (M) signed a peace treaty with the government. During 2008 elections, the CPN (M) won a two-thirds majority but later abandoned their top government positions (Adhikari, 2014).

During the war, the CPN (M)'s People's Liberation Army (PLA) and the Royal Nepal Army conscripted children as soldiers, sentries, spies, cooks, and porters (United Nations, 2006; Human Rights Watch, 2007). Local groups estimate that at the conclusion of the war approximately 9000 members – one-third of the PLA – comprised 14–18 year old with 40% being girls (Human Rights Watch, 2007). An estimated 10% of the Royal Nepal Army during the conflict was below the age of 18 (Singh, 2004).

Our prior research demonstrated that child soldiers in Nepal showed a higher burden of depression, PTSD, and functional impairment than civilian children who were not associated with armed groups at the conclusion of the war (Kohrt *et al.* 2008). In 2007, a few months after signing peace accords, 55% of child soldiers had PTSD compared with 20% of matched civilian children (Kohrt *et al.* 2008).

While differences in war trauma exposure (i.e. the greater exposure among child soldiers compared with civilian children) explains some of the difference between child soldiers and civilian children, there was still a significant difference between the two groups even after controlling for war-related exposures (Kohrt *et al.* 2008). After the war, discrimination against child soldiers in their homes, schools, and communities contributed to mental health and psychosocial problems (Kohrt *et al.* 2010*a*, *c*). This is consistent with findings regarding stigma and discrimination affecting mental health of former child soldiers in other conflicts (Betancourt *et al.* 2013). Reintegration packages represent one commonly used approach to improve former child soldiers’ education, economic, and psychosocial status, as well as a technique used in hopes of decreasing re-recruitment into armed groups or involvement in other violent activities.

### Reintegration packages

In 2007–2008, Transcultural Psychosocial Organization (TPO) Nepal conducted a study of child soldiers before and after participating in psychosocial and reintegration services provided by UNICEF Partner NGOs in eight districts of Nepal (Kohrt *et al.* 2010*b*). Child soldiers in program districts could select among different reintegration packages. The main packages were education, vocational training, apprenticeship, and income-generating activities.

*Education reintegration packages* (ERPs) were designed to address the lost years of education experienced by child soldiers during their association with an armed group. In Nepal, education reintegration programs were supported through block grants to schools or materials support through School Management Committees, i.e. individual children and families were not given money to send child soldiers back to school. Funds provided in block grants can be used to waive tuition fees for former child soldiers, to buy supplies for target children such as stationery and uniforms, and to support activities such as hiring new teachers and building classrooms to address the influx of children in post-war context. In addition, educational grants can be used to design and implement accelerated 6- or 9-month curriculums that allow former child soldiers to master material from multiple grades in a brief period so that they can then mainstream with same-age peers. Without accelerated programs, former child soldiers in their late teens may join classroom cohorts 5–6 years younger than them.

*Vocational training reintegration packages* are an alternative to formal education. Former child soldiers who are unable to meet acceptance standards for schools or choose not to rejoin school select vocational training reintegration packages. Vocational training programs for boys tended to focus on radio repair and for girls the skill was typically tailoring. Local NGOs that facilitated vocational training were encouraged to provide certification of vocational training that may help the child soldiers find employment after the training. Vocational training grants included materials (e.g. sewing machines and radio repair kits), in some programs.

*Apprenticeship reintegration packages* were also available. As opposed to vocational training structured courses, apprenticeship comprised pairing a former soldier with an employer in the community and having them learn a trade. For example, child soldiers entered apprenticeships with truck drivers and other professionals. A reintegration programs subsidized these learning and training opportunities by providing a stipend to the trainee and materials for teaching to the master trainer. Apprenticeship and on-the-job training should reflect the local market demand to ensure sustainability.

*Income generation reintegration packages* were provided in instances where former child soldiers had an opportunity to pursue a business endeavor and required start-up funds in the form of micro-grants or loans. For example, some former child soldiers received micro-grants to raise poultry or goats. Others used micro-grants to start small businesses such as roadside stalls for tea and household goods.

Each child soldier participated in only one reintegration package. Those in school did not participate in the vocational training, apprenticeship, or income-generating programs. Selection of reintegration packages was based on preferences reported by the child soldier. From an evaluation standpoint, this thus represented an equipoise condition in which beneficiaries selected among interventions available to them. In addition to participation in one reintegration package, all child soldiers were in communities with post-war psychosocial support programs that were made available to both former child soldiers and other children impacted by war. For a description of the psychosocial program, see Kohrt *et al.* (2015).

Our objective was to evaluate the impact of the reintegration packages on mental health. Specifically, we were most interested in evaluating the impact of the formal ERP, which was the most commonly selected package, *v.* no reintegration package on mental health. We were also interested in evaluating the impact of ORPs (i.e. vocational, apprenticeship, and income generation) *v.* no reintegration package on mental health outcomes. We hypothesized that the ERP would have the largest effect on mental health outcomes, specifically on depression. We hypothesized this because our qualitative work suggested that education was the most prioritized resource of returning child soldiers (Morley & Kohrt, 2013); moreover, referral to programs such as vocational training was often seen as secondary option for former child soldiers who were assumed by parents or NGO staff to not be capable of succeeding in school (Adhikari *et al.* 2014). Therefore, education was expected to be the highest priority support and have the greatest benefit to those who could obtain the package from a mental health and psychosocial perspective.

However, there are a number of pathways in which mental health may have influenced selection choice and participation that would bias interpretation of mental health outcomes. In prior samples of child soldiers in Nepal, low educational achievement prior to reintegration associated with poor mental health outcomes (Kohrt *et al.* 2008). Because limited prior educational achievement may bias against participation in the formal education reintegration program, the impact of education may be over-estimated in standard comparisons. Other factors such as low caste and female gender are associated with poor mental health outcomes (Kohrt *et al.* 2008, 2010*a*). These demographic characteristics are also associated with educational barriers in Nepal. Thus, numerous socioeconomic and demographic factors may associate with poor mental health and lack of participation in ERPs. One way to address such potential confounding factors is by using propensity score weighting to account for differences in the distribution of these demographic characteristics between those who received the ERP compared with those who did not.

### Sample size

The sample size for this study was 258 child soldiers who were all offered enrollment in their choice of reintegration packages, in addition to being offered psychosocial support activities provided by TPO-Nepal and partner NGOs. Community-wide psychosocial support activities (e.g. awareness raising, psychosocial support for teachers, and non-religious child activities) were delivered in all target communities [see Kohrt *et al.* (2015)] regardless of individual former child soldiers’ decisions to participate in specific reintegration packages. All subjects were included in subsequent analyses. Missing data (less than 15% on all variables and less than 6% on all variables except 3) was imputed using single imputation in the Amelia II package in R, which utilizes random sampling from a distribution calculated from observed values on each variable (Honaker *et al.* 2011). Table 1 indicates the baseline characteristics observed in the full sample, prior to and after weighting.

**Table 1.** Means and maximum ASMDs of covariates before and after weighting

Treatments/baseline characteristics	Education package (*n* = 141) (mean or %)	Other package (*n* = 59) (mean or %)	No treatment (*n* = 58) (mean or %)	Maximum ASMD^a^ before weighting	Maximum ASMD^a^ after weighting
% Female	32.7%	50.1%	39.7%	0.37	0.15
Age (years)	15.3	16.0	15.8	0.52	0.13
Caste (*Dalit*)	19.9%	27.1%	31.0%	0.26	0.13
Total traumas (count)	2.0	2.0	2.4	0.33	0.18
DSRS score	12.9	13.6	13.0	0.15	0.07
CPSS score	17.9	18.4	17.8	0.07	0.05
CFI score	5.9	4.9	5.9	0.21	0.11

ASMD, absolute standardized mean difference; DSRS, depression self-rating scale; CPSS, child PTSD symptom scale; CFI, child functional impairment scale.

a Maximum ASMD is reported as the highest ASMD across all pairwise comparisons (i.e. between treatment groups).

### Independent/treatment variable

Participation in the formal reintegration packages was the independent or treatment variable of interest. We evaluated three treatment conditions: (1) ERPs, (2) ORPs (i.e. vocational, apprenticeship, or income generation), and (3) no treatment. The reintegration packages were implemented by local NGOs partnering with UNICEF and are described in detail above. Participants in the two active treatment arms (ERPs and ORPs) were compared with those who did not participate in any reintegration packages.

### Propensity score variables

Weighting variables were selected based on anticipated and observed association with both the outcome of interest and treatment assignment. Age in years was measured at the time of enrollment in the study. Sex was self-reported as male or female. Total number of traumatic events at baseline was a count of traumatic events derived from previous ethnographic research and had a possible range from 0 to 13 (Kohrt *et al.* 2008). Baseline depression self-rating scale (DSRS) for children, child PTSD symptom scale (CPSS), and child functional impairment (CFI) scale scores measured by self-report at the time of study enrollment were also used as weighting variables. The DSRS for children is an 18-item scale assessing depression symptoms on a ‘Mostly’, ‘Sometimes’, or ‘Never’ scale that corresponded with item scores of 2, 1, or 0 (Birleson, 1981), with a range of possible scores on the DSRS is from 0 to 36. The DSRS has been transculturally translated and validated in Nepal (Kohrt *et al.* 2011). The CPSS (Foa *et al.* 2001) is a 17-item scale corresponding to DSM-IV-TR symptoms of PTSD with a range of 0–68 and has previously been transculturally translated and validated in Nepal (Kohrt *et al.* 2011). The CFI is a 10-item scale (range: 0–30) assessing impairment in daily functional role expectations of children in Nepal (Kohrt *et al.* 2010*a*).

### Outcome variables

The primary outcomes assessed were the changes in DSRS, CPSS, and CFI scores from baseline to 1-year follow-up assessment. That is, positive values represented an increase in depression, PTSD, and functional impairment scores from baseline to follow-up, whereas negative values represented a decrease in symptom and impairment scores.

### Details of PSMs

Propensity scores for the three treatment groups were estimated using the *mnps* (multinomial propensity score) function in the twang statistical package in R (McCaffrey *et al.* 2013; Burgette *et al.* 2014). Propensity score weighting to estimate the average treatment effect (ATE) yielded good balance on the measured covariates (see Table 1) and allowed pairwise comparisons between all three treatment arms (Stuart, 2010). ATE refers to the difference in the outcome comparing a (hypothetical) condition where the whole sample received education (or other) reintegration to a (hypothetical) condition where the whole sample received no reintegration package. We applied the absolute standardized mean difference (ASMD) balance metric as the stopping rule in twang. Weights were assigned by twang to individuals in order to achieve maximum balance across the set of observed covariates in the propensity score model. A given individual's weight is the inverse probability of their being in the treatment group they were in. After this weighting, the covariate distributions in all three groups are more similar to that in the original full sample. The calculated weights were then incorporated into the final outcome models (below).

### Measures of effectiveness of weighting procedures

**Fig. 1.**
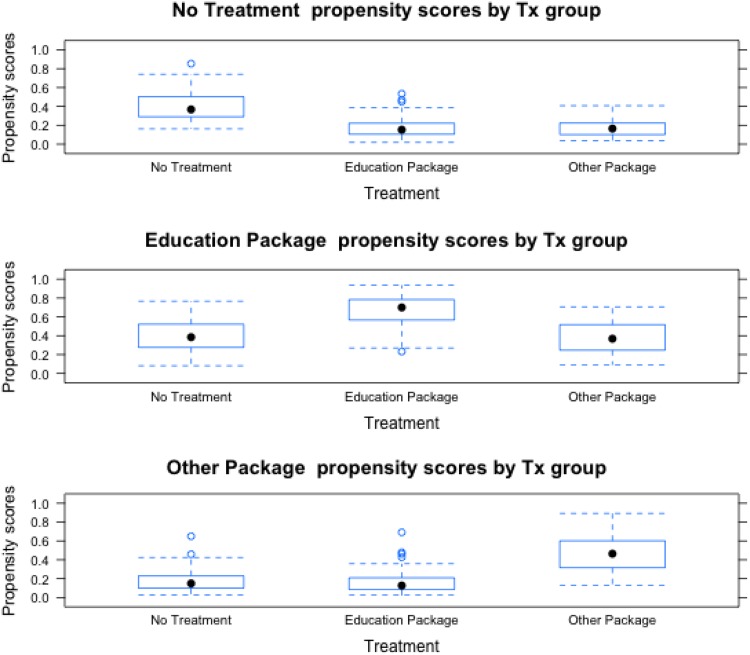
Boxplot illustrating the spread of propensity scores by treatment group for receiving each treatment (i.e. no treatment, education package, and other package). The filled black circles indicate the median propensity score in each treatment group. As the plot demonstrates, there was substantial overlap in the total spread of propensity scores, but the central tendency differed by treatment group.

In order to evaluate the effectiveness of the weighting procedures, we examined the covariate balance in the weighted treatment groups. ASMDs were used to evaluate the similarity of the treatment groups. Both numerical and visual inspection (see Fig. 1 and Table 1) were used to assess covariate balance, with standardized mean differences below 0.25 indicating good balance (Rubin, 2001; Stuart, 2010).

### Estimation of treatment effects

Following weighting procedures (above), treatment effects were estimated using a doubly robust linear regression model with the outcome predicted by treatment group and covariates and fit using the weights defined above.

## Results

Child soldiers in each treatment group differed from comparison child soldiers in other treatment groups (including the no treatment group) on key measured pre-treatment variables. Those receiving the ERPs were less likely to be female or from *Dalit/Nepali* castes and more likely to be younger than those in the other treatment groups (see Table 1). The three groups were generally comparable on measures of traumatic events and all the three pre-treatment clinical scales (i.e. DSRS, CPSS, and CFI). The application of propensity score weighting addressed these imbalances in the distributions of baseline covariates. Figure 1 illustrates the distribution of propensity scores for each treatment group. The figure demonstrates moderate overlap in the propensity scores between treatment groups.

**Fig. 2.**
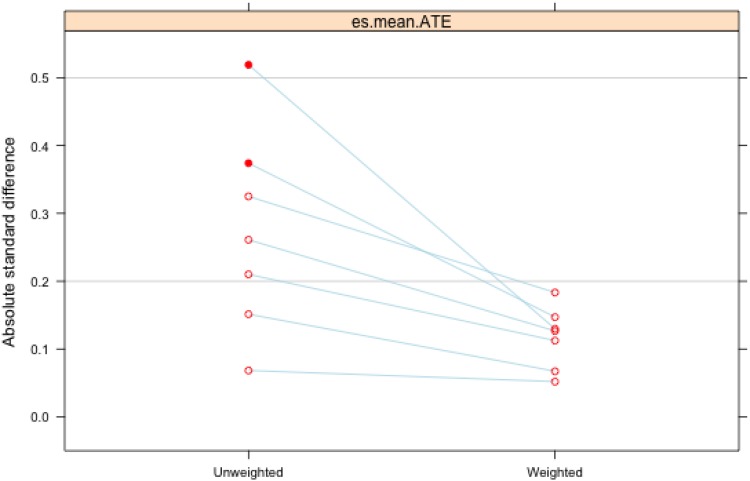
Plot of the maximum absolute standardized mean difference (ASMD) of covariates. Points on the left (‘Unweighted’) represent maximum ASMD (across the three treatment groups) prior to weighting and are connected by lines to the corresponding value of the same covariate's maximum ASMD following weighting on the right (‘Weighted’). Values less than 0.2 indicate adequate balance between treatment and control groups.

Table 1 displays the means and maximum ASMDs of the covariates of interest before and after weighting. As indicated in the columns in the right side of the table, maximum ASMDs were reduced to less than 0.20 for all covariates of interest after weighting, indicating good balance among the observed covariates. Figure 2 graphically displays the change in maximum ASMDs before and after weighting, again indicating that weighting resulted in a substantial improvement in balance, especially for the variables with highest standardized mean differences prior to weighting.

### Estimated treatment effects

The estimated treatment effect of the ERP on depression compared with no treatment (after adjusting for age, sex, caste, traumatic events, and all the three baseline scale scores) was a non-significant reduction in DSRS symptom score by 0.59 (95% CI −1.97 to 0.79) (Table 2). The ERP was associated with a non-significant increase of 1.15 (95% CI −1.55 to 3.86) in the CPSS score and a non-significant increase of 0.91 (95% CI −0.31 to 2.14) on the CFI.

**Table 2.** Treatment effect estimates of ERP and ORPs (v. no treatment) on depression, PTSD, and function impairment

Outcome measure	Education package *v.* no treatment adjusted treatment effect estimate (95% CI)*	ORP *v.* no treatment adjusted treatment effect estimate (95% CI)*
Change in DSRS	−0.59 (−1.97 to 0.79)	−0.60 (−2.16 to 0.96)
Change in CPSS	1.15 (−1.55 to 3.86)	0.66 (−2.24 to 3.57)
Change in CFI	0.91 (−0.31 to 2.14)	1.05 (−0.71 to 2.80)

ERP, education reintegration package; ORPs, other reintegration packages; DSRS, depression self-rating scale; CPSS, child PTSD symptom scale; CFI, child functional impairment scale.

* All multivariate regressions adjusted for age, sex, caste, traumatic events, baseline CPSS score, baseline DSRS score, and baseline CFI score.

The estimated treatment effect of the ORPs on depression compared with no treatment (after adjusting for age, sex, caste, traumatic events, and all the three baseline scale scores) was a non-significant reduction in DSRS symptom score by 0.60 (95% CI −2.16 to 0.96). The ORPs were associated with a non-significant increase of 0.66 (95% CI −2.24 to 3.57) in the CPSS score and a non-significant increase of 1.05 (95% CI −0.71 to 2.80) on the CFI.

## Discussion

In the context of demobilizing and reintegrating former child soldiers into their home communities immediately after war, it was not considered ethical to randomize children to a waitlist condition. Moreover, children were given the option to select the type of reintegration package that they wanted, thus it was also not appropriate to randomize them to a package not of their choosing. Therefore, in this context it was not possible to conduct an RCT of the effects of reintegration programs. Instead, propensity score weighting was used as a technique to compare the effect of reintegration packages *v.* no treatment. We did not find evidence of significant effects on depression, PTSD, or functional impairment for the evaluated reintegration packages compared with no treatment. The findings suggest that compared with no reintegration package, ERPs or ORPs do not result in different outcomes in terms of depression, PTSD, and functional impairment when reintegration packages are conducted in the context of broader community-wide psychosocial support programs (see Kohrt *et al.* (2015)).

We had hypothesized that education would have been the most beneficial package from a MHPS perspective given outcomes of mixed-methods research that pointed towards the desire of most former children to return to school and cross-sectional associations between education attainment and MHPS outcomes (Morley & Kohrt, 2013). Nearly, half (46%) of eligible participants did not elect to enroll in the education package. Uptake was low among older children who may not have returned to school because of age differences with potential classmates (e.g. returning to school at age 16–17 years with classmates in the 12–13 year age range). Uptake was also particularly low among females and members of *Dalit/Nepali* castes, which is to be expected given historic trends creating barriers for girls and *Dalit/Nepali* castes to participate in school. These observations raise some questions about the appropriateness of child-driven selection processes. Some children, especially girls and *Dalit/Nepali* castes, may not have selected education because of anticipated barriers and discrimination. In such cases, it may have been helpful to explore reasons why the former child soldiers selected certain reintegration packages and then address potential barriers to participation in some packages. For example, future studies would benefit from evaluating parents’ perceived value of education as a determinant of uptake of education packages.

An important issue to consider is that our outcomes for this analysis were limited to depression, PTSD, and function impairment. The benefits of different reintegration programs on other life domains such as employment, economic security, physical health, and social status were not evaluated. There may be differential impact on these outcomes based on type of package received. Moreover, this analysis is limited to the 12-month period during which former child soldiers were receiving services. The longer-term difference in outcomes may have varied based on type of reintegration package, but such differences may not have been observable after only 1 year.

Regarding the statistical approach, a key limitation of the PSMs employed in this analysis is that adjustments could only be made for observed confounders. Thus, there may still be bias due to unobserved confounding. Omitting a key (unobserved) covariate can result in confounding if that characteristic was related to both treatment selection and the outcome(s). For example, in our study parents’ perceived value in education may have affected both treatment assignment (i.e. receipt of education package) and the outcomes of interest. Of note, in addition to balancing the distribution of observed covariates, PSMs may also partially address differences in the distribution of unobserved covariates, at least those correlated with the observed covariates (Stuart, 2010).

Our study was characterized by a relatively small sample size, which reduced statistical power to detect small but true differences in outcomes between treatment groups (i.e. Type II error). A larger study may have been better able to detect true differences between treatment options. That said, the magnitude of the changes observed in this study were very small, suggesting that clinical significant improvement may not be observed even with larger sample sizes. Finally, we note that the use of propensity scores in small samples is a relatively underdeveloped topic and deserves further study.

## Conclusion

Alternative approaches to evaluating interventions in humanitarian crises are needed when RCTs are not possible for ethical, logistical, or other feasibility reasons. Propensity scoring weighting is one analytic approach that can be used when relatively large samples are available and there is variation in the type of services received. The technique is only as helpful as the degree to which potential confounders are also recorded. Therefore, if propensity score weighting is a planned analytic approach, then ideally this should be considered at the design stage to assure that a broad range of confounders are assessed. When working with children, potential confounders related to parents and caregivers also should be assessed whenever possible. In the context of this study with child soldiers in Nepal, we were able to demonstrate comparable outcomes for ERPs *v.* other types of services in the context of a broader community-wide psychosocial intervention. Future research is needed to evaluate long-term outcomes of different packages on mental health and psychosocial well-being, as well as the impact in other key domains of life.
